# Multimodality Imaging in Cardiomyopathies with Hypertrophic Phenotypes

**DOI:** 10.3390/jcm11030868

**Published:** 2022-02-07

**Authors:** Emanuele Monda, Giuseppe Palmiero, Michele Lioncino, Marta Rubino, Annapaola Cirillo, Adelaide Fusco, Martina Caiazza, Federica Verrillo, Gaetano Diana, Alfredo Mauriello, Michele Iavarone, Maria Angela Losi, Maria Luisa De Rimini, Santo Dellegrottaglie, Antonello D’Andrea, Eduardo Bossone, Giuseppe Pacileo, Giuseppe Limongelli

**Affiliations:** 1 Inherited and Rare Cardiovascular Diseases, Department of Translational Medical Sciences, University of Campania “Luigi Vanvitelli”, AORN Ospedali dei Colli-Monaldi Hospital, 80131 Naples, Italy; emanuelemonda@me.com (E.M.); g.palmiero@hotmail.it (G.P.); michelelioncino@icloud.com (M.L.); rubinomarta@libero.it (M.R.); cirilloannapaola@gmail.com (A.C.); adelaidefusco@hotmail.it (A.F.); martina.caiazza@yahoo.it (M.C.); fedeverrillo@gmail.com (F.V.); gaetanodiana1991@gmail.com (G.D.); alfredo.mauriello93@libero.it (A.M.); Iavamick@gmail.com (M.I.); gpacileo58@gmail.com (G.P.); 2 Department of Advanced Biomedical Sciences, Federico II University of Naples, 80138 Naples, Italy; losi@unina.it; 3 Department of Nuclear Medicine, AORN Ospedali dei Colli-Monaldi Hospital, 80131 Naples, Italy; marialuisa.derimini@ospedalideicolli.it; 4 Cardiovascular MRI Laboratory, Division of Cardiology, Ospedale Medico-Chirurgico Accreditato Villa dei Fiori, 80011 Acerra, Italy; sandel74@hotmail.com; 5 Unit of Cardiology and Intensive Coronary Care, “Umberto I” Hospital, 84014 Nocera Inferiore, Italy; antonellodandrea@libero.it; 6 Department of Cardiology, Cardarelli Hospital, 80131 Naples, Italy; ebossone@hotmail.com

**Keywords:** hypertrophic cardiomyopathy, left ventricular hypertrophy, multimodality imaging

## Abstract

Multimodality imaging is a comprehensive strategy to investigate left ventricular hypertrophy (LVH), providing morphologic, functional, and often clinical information to clinicians. Hypertrophic cardiomyopathy (HCM) is defined by an increased LV wall thickness not only explainable by abnormal loading conditions. In the context of HCM, multimodality imaging, by different imaging techniques, such as echocardiography, cardiac magnetic resonance, cardiac computer tomography, and cardiac nuclear imaging, provides essential information for diagnosis, sudden cardiac death stratification, and management. Furthermore, it is essential to uncover the specific cause of HCM, such as Fabry disease and cardiac amyloidosis, which can benefit of specific treatments. This review aims to elucidate the current role of multimodality imaging in adult patients with HCM.

## 1. Introduction

Left ventricular hypertrophy (LVH) is characterized by the increase of LV mass in response to different stimuli (physical, biochemical, or genetic) caused by various conditions and diseases [1]. Therefore, the detection of increased LV wall thickness/hypertrophy, potentially associated with heart failure (HF), arrhythmias, and death in the long term, should prompt the research for its underlying cause and prognostic significance [2,3]. 

Hypertrophic cardiomyopathy (HCM) is defined by an increased LV wall thickness not only explainable by abnormal loading conditions [4,5]. It is often a benign condition that does not require treatment; however, some HCM patients develop severe clinical phenotypes and complications, such as sudden cardiac death (SCD), HF, atrial fibrillation (AF), and strokes [6,7,8].

In more than half of patients, mutations in genes encoding for sarcomeric proteins are accountable for the development of the disease [4,9]. However, several other causes (inherited and not inherited) can be responsible for the HCM phenotype in adults (e.g., Fabry disease, cardiac amyloidosis), and their identification can guide the subsequent management [10,11,12,13,14]. 

Multimodality imaging is an important screening tool for identifying LVH and often provides the first clinical suspicion for specific aetiologies, especially when the medical and familiar history is mute. By a stepwise selection of appropriate diagnostic techniques, especially in the field of multimodality imaging, to collect those clinical and instrumental findings (red flags) that may suggest a specific cause, the identification of an underlying systemic disease with a potential tailored treatment becomes more and more achievable in clinical practice.

**Figure 1:**
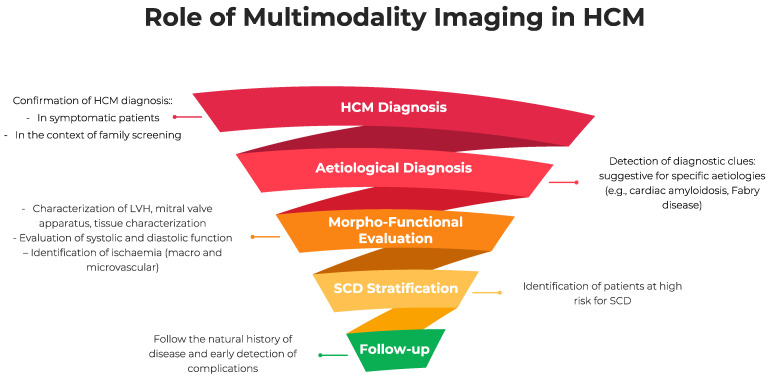
The role of multimodality imaging in hypertrophic cardiomyopathy. LVH, left ventricular hypertrophy; HCM, hypertrophic cardiomyopathy; SCD, sudden cardiac death.

In HCM, multimodality imaging, by different imaging techniques, such as echocardiography, cardiac magnetic resonance (CMR), cardiac computer tomography (CCT), and cardiac nuclear imaging (CNI), provides essential information for diagnosis, sudden cardiac death (SCD) stratification, and management (Figure 1).

This review aims to elucidate the current role of multimodality imaging in adult patients with HCM.

## 2. Echocardiography

Echocardiography, being non-invasive and easily repeatable, has a fundamental role in detecting the different LVH/HCM phenotypes, suggesting disease causes, cardiac morphology and hemodynamic, and disease severity, all steps necessary in clinical practice for the management and treatment strategies. 

### 2.1. Hypertrophic Cardiomyopathy

Echocardiography is a key tool for HCM diagnosis when an unexplained maximum wall thickness (MWT) > 15 mm in any myocardial segment of the LV is evidenced [15]. The asymmetrical pattern is the most common type of LVH, with the involvement of the interventricular septum. However, any pattern of hypertrophy can be described [16]. Near 30% of patients have dynamic left ventricular outflow tract obstruction (LVOTO) caused by the anterior mitral valve’s systolic anterior movement (SAM), and most of them exhibit mitral regurgitation due to poor leaflet apposition with concomitant posteriorly directed jets. Rarely, the obstruction is at the midventricular level [17]. 

Myocardial deformation parameters, such as GLS, are often reduced in pathological hypertrophy despite a normal LVEF, even in carriers who had not developed LVH [18]. 

GLS is typically reduced at the site of LVH. The site of maximal hypertrophy is also and often the site of replacement fibrosis, evaluated by CMR as late gadolinium enhancement (LGE) [19,20]. Moreover, patients with ventricular arrhythmias have worse GLS than those without [21].

Myocardial work (MW), a novel technique for LV systolic assessment derived by speckle-tracking echocardiography, was recently applied in nonobstructive HCM [21,22,23]. Potentially, it could detect the presence of myocardial fibrosis indirectly by the reduced regional myocardial function. However, its role in risk stratification and prognosis needs to be evaluated.

HCM caused by mutations in sarcomeric genes must be differentiated by other conditions which may mimic HCM phenotype but show a different aetiology and treatment [4,5,9,24,25,26,27]. In this paragraph, we will focus on cardiac amyloidosis and Fabry disease, which represent possible causes of non-sarcomeric HCM in adult and elderly patients (Table 1).

### 2.2. Cardiac Amyloidosis

**Figure 2:**
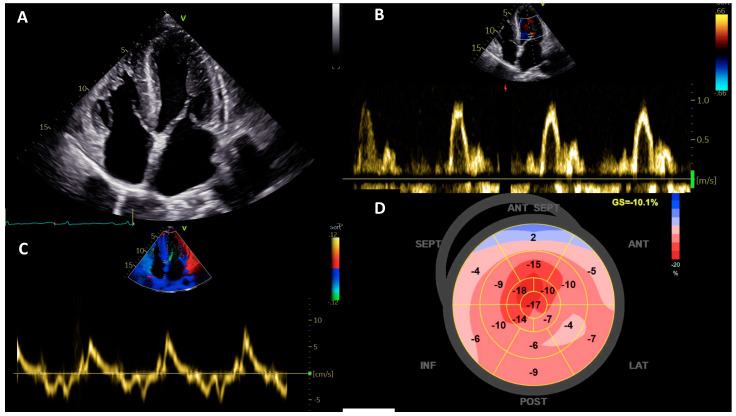
Echocardiographic clues of a patient with AL cardiac amyloidosis (**A**): Apical four-chamber view demonstrates increased biventricular wall thickness with a sparkling texture of the myocardium, thickening of the interatrial septum, and biatrial enlargement. (**B**): Restrictive diastolic pattern. (**C**): Tissue Doppler imaging taken at the septal mitral annulus showing low e’ velocities. (**D**): Global longitudinal strain with the apical sparing pattern.

Cardiac amyloidosis (CA) is caused by extracellular deposition of insoluble fibrils determining multifactorial myocardial damage, LV wall thickening (pseudo-hypertrophy), and consequently, early systolic and diastolic dysfunction leading to heart failure [28]. Both forms of CA, transthyretin-related (ATTR-CA) and immunoglobulin light-chains (AL-CA), share the same phenotype despite fundamental differences in prognosis and treatment. On echocardiography, LVH pattern in CA is more frequently concentric in the presence of a standard or small LV cavity, commonly extended to the right ventricle (RV); LVEF is preserved until the very late stages of disease progression, while the deformation-based parameters detect a significant systolic dysfunction since from the early stages; the left and right atrium are invariably enlarged; diastolic dysfunction is present since from the early stages of the disease with augmented filling pressures and the specific restrictive pattern present predominantly at advanced stages of disease; thickened interatrial septum and mild-to-moderate pericardial effusion are common [10] (Figure 2). 

Several echocardiographic red flags may raise the suspicion of CA. A prospective multicenter Italian study is currently investigating the prevalence of echocardiographic red flags of CA in consecutive patients ≥55 years who performed routine transthoracic echocardiography [29].

Ventricular involvement in CA is characterized by early basal involvement that starts from the subendocardial layers, acquiring a transmural fashion subsequently with mid-ventricular involvement and LV apical sparing, responsible for generating the same pattern using longitudinal strain analysis [30,31]. This pattern is quite specific and valuable in suspected CA patients with concentric LVH [32]. Another important parameter that should raise the suspicion of CA is the ejection fraction on strain ratio (EFSR), which comes from the combined evaluation of the LVEF and GLS. The evidence of the presence of LVEF associated with a significant reduction in GLS is typical of CA since the early stage of the disease. Compared with other classical and deformation-based parameters, this parameter provides high sensitivities and specificities in differentiating CA from other hypertrophic substrates [33]. Strain imaging parameters also have prognostic significance [34]. Furthermore, LV twist, assessed by speckle tracking echocardiography, was suggested providing additional pathophysiological and clinical insights for different cardiomyopathies [35]. 

Many diagnostic scores were recently proposed to improve CA early diagnosis in patients with unexplained LVH. A multicenter study has recently developed two different multiparametric echocardiographic scores to suggest the diagnosis of CA. The first was formulated to detect myocardial involvement in systemic AL amyloidosis, and the second was to facilitate CA diagnosis in patients with increased LV wall thickness (IWT). The score is based on four variables (relative wall thickness (RWT), E/e’ ratio, GLS, tricuspid annular plane systolic excursion (TAPSE)) for AL-CA patients and five variables for IWT (the previous with the addition of septal apical-to-base ratio) [36]. Monda et al. have recently validated the IWT score in an independent population and confirmed its diagnostic value [37]. Another multiparametric echocardiographic score, the AMYLoidosis Index (AMYLI), is based on the product of two variables (RWT and E/e’ ratio), and a value < 2.22 excludes CA with sufficient sensibility and specificity [38]. Recently, the role of MW analysis was also investigated in CA patients. Of interest, MW may be of prognostic value [39,40,41]. A recent observation from our group has also highlighted the possibility to differentiate between CA and other causes of LVH and between AL and ATTR-CA by MW parameters [42]. 

However, these results were based on a small cohort; indeed, further investigation is needed.

### 2.3. Fabry Disease

FD is an X-linked multisystemic lysosomal storage disorder caused by the deficiency of the α-galactosidase enzyme, thus resulting in an accumulation of glycosphingolipids within several tissues [12], mainly represented by the heart, brain, and kidneys. 

Patients with FD cardiomyopathy show severe concentric LVH. The development of replacement fibrosis, commonly identified in the basal segment of the posterolateral wall, and the afterload changes related to FD vasculopathy, are responsible for an increase in LV volumes, which lead to a more spherical shape [43,44,45]. The replacement fibrosis can be indirectly evaluated using myocardial deformation imaging [46]. GLS in the basal posterolateral wall is significantly decreased in FD patients and could represent an indirect sign of myocardial replacement fibrosis [47]. Moreover, a specific LV deformation pattern in patients with FD was recently described, characterized by the loss of normal circumferential strain base-to-apex gradient [48]. This pattern can be found in FD patients regardless of LVH status, native T1 values and LGE at CMR, potentially representing an early marker of cardiac involvement in FD [49]. 

The glycosphingolipids accumulation in FD predominantly involves the subendocardial ventricular layers. Using layer-specific strain imaging, it was evidenced that the GLS reduction was mainly the result of the involvement of subepicardial strain, which is responsible for the increase in longitudinal strain myocardial gradient [50,51].

## 3. Cardiac Magnetic Resonance

Cardiac magnetic resonance (CMR) plays an essential role in diagnosing and managing patients with HCM [4,5] or other cardiomyopathies [52,53]. It provides information on the ventricular morphology, function, and magnetic properties, other than the presence and localization of fibrosis. The evaluation of these parameters is helpful in different settings, such as diagnosis, risk stratification, and differential diagnosis.

### 3.1. Hypertrophic Cardiomyopathy

According to the current guidelines, CMR should be considered at the baseline assessment of each patient with suspected or confirmed HCM, if available [4,5]. It comprehensively evaluates both ventricles with a high spatial resolution [53,54,55] and represents an essential tool in the measurement of the LV maximal wall thickness (MTW) and mass in patients with inadequate acoustic windows or when some ventricular regions cannot be adequately evaluated with echocardiography [55,56]. In addition, CMR has a high sensibility in detecting LV apical hypertrophy [57], aneurysms [58], myocardial crypts, and papillary muscle abnormalities [59,60].

CMR can also evaluate the presence of myocardial fibrosis. Indeed, LGE can be found in about two-thirds of patients [61]. Two major distribution patterns were evidenced in sarcomeric HCM: patchy intramural LGE within the hypertrophied segments; right ventricular (RV) insertion point LGE [62]. It was proposed that the former corresponds to replacement fibrosis and the latter to interstitial fibrosis or myocyte disarray [63]. Of importance, the presence of LGE was associated with an increased risk of adverse outcomes for patients with HCM, including SCD, and the risk is proportional to the extent of LGE [64,65,66]. 

Thus, CMR is crucial to properly perform the SCD risk stratification of patients with HCM [4,5]. In the context of the SCD risk stratification, it can be helpful:

- to identify the presence of apical aneurysms, defined as a discrete thin-walled dyskinetic or akinetic segment of the distal portion of the LV chamber, which represents a significant risk factor for SCD [67];
- to measure the MWT of the LV, in particular in patients with poor echocardiographic acoustic windows, keeping in mind that this parameter is linearly associated with increased SCD and a value of ≥30 mm is a significant risk factor for SCD [15,68];
- to detect patients with systolic dysfunction (LV ejection fraction < 50%), the “end-stage” phase of HCM, associated with poor outcomes and increased risk for SCD [69];
- to describe the presence and the extent of LGE, with a threshold of ≥15% defined as associated with an increase in SCD risk [65,66].

However, while the first three parameters are recognized as strong risk factors for SCD and in their presence an ICD implantation should be considered, less clear is the management of patients with extensive LGE and no other risk factors. Therefore, the incorporation of LGE into the risk assessment for SCD in patients falling into the “grey zone”, such as those with an intermediate HCM risk score (between 4% and 6%) or without major AHA/ACC risk factors, it is reasonable [4,5]. 

In the context of multimodality imaging, the role of machine learning and artificial intelligence is gaining attention. Among others, possible applications in HCM are the evaluation of LV myocardial fibrosis [70] and MWT [71], two important risk factors for SCD. Indeed, when an indication for ICD implantation is only based on the MWT, the weight of measurement errors and inter-observer variability should be considered, and an expert evaluation should be performed. However, it was recently evidenced that a machine learning algorithm was superior to human experts in measuring MWT, with significant implications for diagnosis and risk stratification [71].

CMR should be considered when planning a surgical myectomy, especially if the transesophageal acoustic windows are suboptimal and in patients with multi-level or biventricular outflow obstruction [4].

Recently, the value of T1 and T2 mapping in HCM was investigated. It was found that native T1 and T2 values were significantly elevated in hypertrophic and even non-hypertrophic segments, suggesting that tissue remodeling may precede the morpho-functional changes of the LV [72]. Moreover, native T1 values showed elevated values, even in the absence of LGE, and were correlated with the extent of the LV mass, suggesting that HCM patients may have increased interstitial fibrosis despite the lack of LGE [73,74].

Finally, the role of CMR is gaining attention for its potential use in evaluating myocardial stiffness, which plays a crucial role in the diastolic function of the LV. In HCM, myocardial stiffness and, of consequence, diastolic dysfunction is mainly caused by fibrosis and myocyte disarray. The non-invasive evaluation of myocardial stiffness is complex in clinical practice, and it is generally indirectly evaluated by assessing biomarkers (e.g., B-type natriuretic protein) and echocardiographic parameters (e.g., diastolic pattern or E/e’). CMR offers the possibility to investigate the two major determinants of myocardial stiffness in HCM, such as the myocardial fibrosis and the collagen volume fraction, using LGE and ECV, respectively [75,76]. Furthermore, CMR can non-invasively evaluate the myocardial stiffness using magnetic resonance elastography (MRE) [77] and four-dimensional (4D) flow [78]. MRE is a phase-contrast magnetic resonance imaging that estimates stiffness using shear waves [77], while 4D flow CMR offers a new way to assess the intraventricular diastolic flow and non-invasively evaluate the myocardial stiffness with greater accuracy and less interobserver variability compared to echocardiographic evaluation [78]. Although not currently used in clinical practice, MRE and 4D flow CMR represent emerging techniques with diagnostic and prognostic purposes.

### 3.2. Fabry Disease

Several diagnostic clues suggestive for specific causes of HCM (e.g., cardiac amyloidosis, Fabry disease) should be identified using CMR, and their presence can guide the subsequent investigation [9,76,79]. The identification of LGE with a mid-wall distribution in the basal segment of the inferolateral wall is typical of FD, and it may appear before LVH, especially in female patients [12,80]. Tissue characterization using T1 mapping is gaining an essential role in the differential diagnosis of patients with LVH. According to the analysis of native T1 values, the presence of LVH and LGE, three stages of cardiac involvement are described in Fabry disease. The first stage is characterized by normal or low native T1 values, caused by the intracellular glycosphingolipid accumulation, without LVH. The second stage shows a low native T1 value presence of LGE with or without LVH. In the third stage, the native T1 values exhibit pseudo-normalization due to extensive LGE [81]. 

### 3.3. Cardiac Amyloidosis

Cardiac amyloidosis produces a unique pattern of subendocardial LGE, or, in rare cases, the distribution of LGE may be patchy, diffuse, or transmural [82]. In cardiac amyloidosis, the extracellular amyloid infiltration is responsible for elevated native T1 values [10,28,83], and its demonstration can be especially useful in patients with severe renal dysfunction, where gadolinium contrast should be avoided. In addition, T1 mapping can be used to estimate the myocardial ECV fraction, which represents a surrogate to quantify amyloid burden [84]. Patients with cardiac amyloidosis exhibit increased ECV fraction, which correlates with different echocardiographic parameters, such as LV mass, septal thickness, systolic volume, and left area. Moreover, it can also be found in those cases without LVH and when LGE does not demonstrate evidence of cardiac amyloidosis, thus resulting as a sensitive test for the early detection of cardiac involvement in patients with amyloidosis [85]. Other rare causes of LVH, such as metabolic or neuromuscular, may exhibit peculiar CMR patterns [85,86]; however, they are more common in paediatric patients [86,87] while rarely representing a cause of HCM in adults.

## 4. Coronary CT Angiography and Nuclear Imaging

### 4.1. Coronary Artery Disease in Hypertrophic Cardiomyopathy

Although HCM patients with chest pain have shown a lower incidence of epicardial coronary artery stenosis compared with the risk-adjusted general population [88], severe CAD is a predictor of adverse clinical events [89]. Nevertheless, data from large RCTs [90,91] have shown conflicting results regarding the benefit of reperfusion compared with medical therapy [92].

Non-invasive multimodality imaging may be useful to exclude obstructive CAD, particularly in specific clinical settings, as among patients with chest pain, unexplained left ventricular systolic dysfunction, or in case of disproportion between the magnitude of dyspnoea and heart failure symptoms. However, non-invasive studies are limited by the high prevalence of microvascular disease in HCM, accounting for low predictive positive value (PPV), according to pre-test probability and clinical presentation [93].

Echocardiography with high-dose dipyridamole (0.84 mg/kg iv in 6 min) is used to assess regional wall kinesis and coronary flow reserve (CFR) response during stress. The combination of a new stress-induced wall-motion abnormality and reduced CFR velocity on the LAD reaches high diagnostic accuracy to identify CAD [94].

SPECT with Tc99 or Tl-201 may be performed in patients with a low pre-test probability of CAD [95,96]. However, perfusion defects can be detected even in the absence of epicardial CAD and, because of higher uptake in hypertrophic segments, relatively abnormal perfusion can be found in non-hypertrophied regions. 

Unlike SPECT, PET with N13-labelled ammonia or O15-labelled water allows a quantitative measurement of the absolute myocardial blood flow; however, false-positive results are common in the presence of significant myocardial fibrosis or in cases of severe hypertrophy [97,98]. Coronary CT angiography (CCTA) has adequate diagnostic accuracy in identifying epicardial CAD in HCM patients, showing lower rates of obstructive CAD among symptomatic HCM patients compared with the general population (7% vs. 33% *p* < 0.001) and significantly lower false-positive rates compared with SPECT or PET [84]. Given the high incidence of myocardial bridges in HCM, CCTA may be useful to assess coronary anatomy [99]. However, the role of myocardial bridges remains unclear, and observational studies failed to demonstrate their association with sudden death [100].

In conclusion, CCTA may be the preferred imaging technique in patients with a low pre-test probability of CAD, whereas coronary angiography remains the cornerstone in patients with high pre-test probability or when non-invasive studies show unequivocal findings.

### 4.2. Cardiac Amyloidosis

The suspicion of cardiac amyloidosis may be triggered by some relevant clinical scenarios, such as low-flow low-gradient aortic stenosis, HFpEF, or when increased LV wall thickness (>12 mm) is associated with extracardiac red flags [10,29,101]. Cardiac scintigraphy with Tc99m-bone-avid radiotracers (PYP, HMDP or DPD) is highly specific in detecting transthyretin cardiac amyloidosis (ATTR-CA) [102,103]. Nevertheless, grade 2 or 3 myocardial uptake of Tc99m-PYP/DPD/HMDP was demonstrated in almost 20% of patients affected with AL amyloidosis [104]. Semiquantitative evaluation, based on Perugini score or heart/contralateral lung uptake (H/CL) ratio>1.5 at 1h, has high accuracy for the diagnosis of ATTR-CA [105,106,107,108]. Given the high prevalence among the elderly of monoclonal gammopathy of uncertain significance (MGUS), the diagnosis of ATTR-CA requires the combination of positive bone scintigraphy, showing Perugini grade 2 or 3 myocardial uptake, and the absence of monoclonal chains demonstrated by means of serum and urine immunofixation or free light chain assay [104].

False-positive results for bone scintigraphy may be associated to AL amyloidosis, hydroxychloroquine toxicity, or apolipoprotein amyloidosis. On the other hand, particular conditions, such as rib fractures, technical errors, and recent myocardial infarction, may mask an underlying positive bone scintigraphy. Histological confirmation by means of endomyocardial biopsy may be required in case of non-concordant findings and when the clinical presentation is highly suspicious for an underlying ATTR-CA [101]. 

There is compelling evidence to demonstrate the usefulness of molecular imaging based on amyloid-binding PET tracers (C11 Pittsburgh compound, F18-Florbetapir, and F18-Flourbetaben) to diagnose cardiac amyloidosis, to quantify the extent of amyloid infiltration, and to detect molecular changes in their composition, thus being potential markers of therapeutic response [109,110,111]. In a case-control study, Dorbala et al. have demonstrated that 18F-Fluorbetapir PET scans were useful to differentiate cardiac amyloidosis from healthy subjects; however, the investigators did not conduct a prespecified analysis to differentiate AL from ATTR deposition and did not extend their scan acquisition beyond 30 min [112]. Recently, Genovesi et al. demonstrated that late static18F-Fluorbetaben PET scans might be helpful to discriminate cardiovascular involvement due to AL amyloidosis from ATTR amyloidosis [113]. The time course of myocardial uptake seems to suggest that the difference between AL and ATTR amyloidosis remains significant within 20–60 min for radiotracer injection. However, larger studies are needed to assess the role of molecular imaging to differentiate the amyloid subtype and therapeutic response.

### 4.3. Fabry Disease

Many observational studies have investigated the use of I123-metaiodobenzylguanidine (MIBG) scintigraphy to assess the extent of autonomic dysfunction in Anderson–Fabry disease (FD). Decreased MIBG uptake seemed to correlate with the extent of fibrosis diagnosed by means of late gadolinium enhancement (LGE), although autonomic dysfunction seemed to precede myocardial fibrosis [114]. On the other hand, among patients with FD, abnormal myocardial perfusion was demonstrated both on SPECT and PET, in the absence of coronary artery disease, and interestingly, no improvement in myocardial blood flow was observed after 12 months of enzyme replacement therapy (ERT) [115,116]. More recently, 18FDG PET was used, in combination with CMR, to detect early cardiac involvement in FD, demonstrating increased radiotracer uptake and a trend towards pseudo-normalization of abnormal T1 mapping values suggesting that inflammation may be the preclinical phase of cardiac involvement in FD [117,118].

## 5. Conclusions

Multimodality imaging is crucial to first confirm the diagnosis of HCM and then to accurately evaluate features associated with high-risk of SCD. In addition, proper identification of patients with specific causes of HCM is crucial to start appropriate treatment. While echocardiography remains the first-line technique for the assessment of patients with HCM, the role of CMR, CCT, and CNI is expected to increase in the future, given their importance in providing some details nonevaluable by echocardiography alone.

## Figures and Tables

**Table 1:** Echocardiographic features of the different causes of left ventricular hypertrophy in adults.

Type of LVH	Echocardiography	Other Red Flags
Athlete’s heart	2D: eccentric LVH with MWT < 14 mm and preserved LVEF and TDI velocities. Normal or supernormal systolic and diastolic dysfunction. Strain imaging: preserved GLS with increased transverse, radial and circumferential strain.	History of intense physical exercise.
Hypertrophic cardiomyopathy	2D: Asymmetrical hypertrophy with MWT > 15 mm; possible apical LVH; presence of LVOTO and/or SAM with secondary MR with posteriorly directed jet and/or apical aneurysm. Strain imaging: reduced longitudinal strain and constructive work at ventricular MWT level.	History of sudden cardiac death. ECG: voltage criteria for LVH, biatrial enlargement and ST-T abnormalities.
Arterial hypertension	2D: Asymmetrical hypertrophy with prevalence for basal interventricular septum and preserved LVEF. Strain imaging: reduced peak systolic strain/strain rate at basal interventricular septum; progressive GLS reduction.	History of arterial hypertension.
Cardiac amyloidosis	2D: Concentric LVH with preserved LVEF and progressive reduction in LV volumes; biventricular involvement; early diastolic dysfunction with biatrial enlargement; pericardial effusion. Strain imaging: Relative apical sparing pattern with increased EFSR; reduced GCW and GWE by LVMWI.	Extracardiac involvement: neurological (peripheral neuropathy, carpal tunnel syndrome, autonomic neuropathy, spinal cord stenosis), ocular (vitreous opacity, cataract) ECG: voltage discordance pattern (increased LV mass at cardiovascular imaging with normal or reduced QRS voltages); pseudoinfarction pattern; AF; conduction abnormalities.
Fabry disease	2D: Predominant severe concentric LVH with preserved LVEF and progressive increase in LV volumes. Strain imaging: Reduced longitudinal strain in the basal posterior-lateral wall; impaired LV subendocardial longitudinal strain at multilayer strain analysis.	ECG: short PR, conduction abnormalities. Extracardiac involvement: cutaneous (angiokeratoma, hypohidrosis), neurological (acroparaesthesiae, stroke-like events), renal (proteinuria, ned-stage kidney failure), ocular (corneal dystrophy), gastrointestinal (abdominal pain, vomiting, diarrhoea).
Aortic stenosis	2D: Concentric LVH with preserved LVEF, valvular leaflet calcification with decreased opening, increased transaortic valve pressure gradient. Strain imaging: Reduced GLS (predominantly at LV basal level); reduced LV basal rotation with preserved LV apical rotation and increased LV torsion.	Paradoxical low-flow pattern in elderly males with neurological involvement (e.g., carpal tunnel syndrome, spinal cord stenosis, peripheral neuropathy) is suggestive of CA.

2D, Two-dimensional; AF, Atrial Fibrillation; CA, Cardiac Amyloidosis; EFSR, Ejection Fraction on Strain Ratio; ECG, electrocardiography; GCW, Global Constructive Work; GLS, Global Longitudinal Strain; GWE, Global Work Efficiency; LVEF, Left Ventricular Ejection Fraction; LVH, Left Ventricular Hypertrophy; LVMWI, Left Ventricular Myocardial Work Indices; LVOTO, Left Ventricular Outflow Tract Obstruction; MR, Mitral Regurgitation; MWT, Maximal Wall Thickness; SAM, Systolic Anterior Movement of Mitral Valve; TDI, Tissue Doppler Imaging.
